# Chronic black tea extract consumption improves endothelial function in ovariectomized rats

**DOI:** 10.1007/s00394-015-1012-0

**Published:** 2015-08-15

**Authors:** Fung Ping Leung, Lai Ming Yung, Ching Yuen Ngai, Wai San Cheang, Xiao Yu Tian, Chi Wai Lau, Yang Zhang, Jian Liu, Zhen Yu Chen, Zhao-Xiang Bian, Xiaoqiang Yao, Yu Huang

**Affiliations:** Clinical Division, School of Chinese Medicine, Hong Kong Baptist University, Hong Kong, China; Department of Medicine, Brigham and Women’s Hospital, Harvard Medical School, Boston, MA USA; Institute of Vascular Medicine and Li Ka Shing Institute of Health Sciences, Chinese University of Hong Kong, Hong Kong, China; Food and Nutritional Sciences Programme, School of Life Sciences, The Chinese University of Hong Kong, Hong Kong, China

**Keywords:** Black tea, Oxidative stress, Endothelial dysfunction, Aorta, Postmenopause, Theaflavins

## Abstract

**Purpose:**

Menopause escalates the risk of cardiovascular diseases in women. There is an unmet need for better treatment strategy for estrogen-deficiency-related cardiovascular complications. Here we investigated the impact of chronic black tea extract (BT) consumption on cardiovascular function and lipid metabolism using a rat model of estrogen deficiency.

**Methods:**

Female Sprague–Dawley rats were ovariectomized (OVX) and treated with BT (15 mg/kg/day, 4 weeks; active ingredients: theaflavins) or estrogen (E2) treatment for 4 weeks. Serum was collected for measuring cholesterol, triacylglycerol and estradiol levels. Changes in vascular reactivity were examined. The protein levels of NADPH oxidases were assessed by Western blotting. Reactive oxygen species (ROS) level was measured using dihydroethidium fluorescence imaging. The concentrations of cGMP were measured using ELISA kit.

**Results:**

Aortic rings from control, BT-treated and E2-treated OVX rats exhibited a greater increase in Phe-induced contraction after inhibition of NO synthase compared with those from OVX rats. ACh-induced endothelium-dependent relaxations were augmented in aortae and renal arteries in BT/E2-treated OVX rats than in OVX rats. BT/E2 treatment improved flow-mediated dilatation in small mesenteric resistance arteries of OVX rats. BT/E2 treatment restored the eNOS phosphorylation level and reversed the up-regulation of NADPH oxidases and ROS overproduction in OVX rat aortae. ACh-stimulated cGMP production was significantly elevated in the aortae from BT- and E2-treated rats compared with those from OVX rats. BT/E2 treatment reduced circulating levels of total cholesterol.

**Conclusions:**

The present study reveals the novel benefits of chronic BT consumption to reverse endothelial dysfunction and favorably modifying cholesterol profile in a rat model of estrogen deficiency and provides insights into developing BT as beneficial dietary supplements for postmenopausal women.

## Introduction

Cardiovascular disease (CVD) risk rises with age, but for women symptoms can become more manifest after the onset of menopause. In this natural state of estrogen deficiency, hormone replacement therapy (HRT) is effective in reducing the incidence of coronary heart disease as well as in mortality from CVD [1, 2]. However, HRT is also associated with an increased risk of cancers and cardiovascular events, such as venous thromboembolism and stroke [3–5]. Moreover, the results of two large randomized prospective findings disprove the claims of cardiovascular benefits of HRT based on observational studies. The Heart and Estrogen/Progestin Replacement Study was a randomized clinical trial designed to test the efficacy of HRT in the secondary prevention of coronary heart diseases. The results indicated that treatment with oral conjugated equine estrogen plus medroxyprogesterone acetate did not lower the overall rate of coronary heart disease events in postmenopausal women with established coronary disease. In contrast, the treatment increased the rate of thromboembolic events and gall bladder disease [6]. Another prospective study of primary prevention of CVD, named as the Women’s Health Initiative (WHI), was stopped in early phase, as it was demonstrated that overall health risks exceeded benefits from use of combined estrogen plus progestin for an average 5.2-year follow-up among healthy postmenopausal US women [7]. Another study evaluated the results of the therapeutic arm of the WHI investigating the isolated use of conjugated estrogens versus placebo among hysterectomized postmenopausal women. There was an increased risk of cerebral vascular accident, but a reduced risk of hip fracture and null effect on the incidence of CVD, as well as potential reduction in the incidence of breast cancer. Overall, the risk–benefit index was neutral [8].

Tea, the most popular beverage worldwide, is consumed in three basic forms: green tea, black tea and oolong tea. Recently, there has been a mounting interest in understanding the cardiovascular and metabolic benefits of polyphenolic flavonoids in tea, which can be used as health supplement. Compelling evidence in human and murine models also suggests various cardioprotective benefits of consuming tea or tea polyphenols under pathological conditions, e.g. hypertension, atherosclerosis, diabetics, hypercholesterolemia, obesity, and are attributed to antioxidative, anti-thrombogenic, anti-inflammatory, hypotensive and hypocholesterolemic properties of tea polyphenols [9, 10]. Yung et al. [11] also discussed the cardiovascular benefits of tea polyphenols in four related fields (1) vasorelaxant effect; (2) protective effect against endothelial dysfunction; (3) antioxidant effect and (4) hypolipidemic effect [11]. A recent meta-analysis suggests that daily consumption of black tea equaling three cups per day could prevent the onset of ischemic stroke [12]. Black tea is equally potent as green tea in promoting cardiovascular health, as theaflavins and thearubigins predominantly counterbalance the lack of catechins in black tea [13]. Overall, tea represents a promising tool for the prevention of cardiovascular complications. In the present study, we test the hypothesis that black tea consumption ameliorates endothelial dysfunction during estrogen deficiency.

## Methods

### Animal treatment and artery preparation

All the experimental protocol was approved by the institutional animal care and use committee and was consistent with the Guide for the Care and Use of laboratory Animals published by the National Institutes of Health. Adult female Sprague–Dawley rats (3 months old, weighing 200–230 g) were purchased from the Laboratory Animal Service Center, Chinese University of Hong Kong. Rats were anesthetized using sodium pentobarbital (40 mg/kg body weight, intraperitoneal injection), ovariectomized via a mid-abdominal route. Three months after ovariectomy, the rats were randomly assigned into three experimental groups: (1) OVX, ovariectomized rats; (2) OVX + BT, ovariectomized rats receiving daily administration of black tea extract (BT) (Quality Phytochemicals LLC; Product code: QP-Black tea extract; Source: *Camellia sinensis* O. Ktze; Active ingredients (assay by HPLC): Theaflavins 70 %) at 15 mg kg-1 day-1 by gastric gavage for 4 weeks; and (3) OVX + E2, ovariectomized rats with estrogen (E2) treatment for 4 weeks, by inserting a 17β-estradiol pellet (0.5 mg/pellet, Innovative Research of America) into the dorsal subcutaneous pockets [14]. The sham-operated rats served as controls (Control). Black tea extract administration was initiated at the fourth week after ovariectomy. The animals were killed at the age of 7 months. All animals were housed under conditions of controlled temperature (23 ± 2 °C) and humidity (60 %) with 12-h light/dark cycles.

### Blood collection, measurement of cholesterol profiles, and serum estrogen

At the end of chronic treatment, the rats were euthanized by CO_2_ suffocation and sera were collected for analysis of levels of estrogen and lipids. Hearts and uteri were dissected free of surrounding fat pads and then weighed. Serum levels of total cholesterol (Sigma 352-20), triacylglycerols (Sigma 336-20), and HDL cholesterol were determined using enzymatic kits as described previously [14]. Serum 17β-estradiol levels were measured using an estradiol EIA Kit (Cayman Chemical).

### Artery preparation

The thoracic aorta was dissected and cleaned of adhering connective tissue in ice-cold and oxygenated Krebs-Henseleit solution containing (mM): 119 NaCl, 4.7 KCl, 2.5 CaCl_2_, 1 MgCl_2_, 25 NaHCO_3_, 1.2 KH_2_PO_4_, and 11 d-glucose. Each aorta was cut into several ring segments (3 mm in length) for parallel studies, and each experiment was performed on rings obtained from different rats. The fresh aortic ring was suspended between two stainless steel hooks in a 10-mL organ bath filled with Krebs solution. Bathing solution was continuously bubbled with 95 % O_2_ and 5 % CO_2_ and maintained at 37 °C (pH of 7.3–7.5). An optimal baseline tone of 2.5 g was applied to all rings. The renal interlobar arteries were cleaned of adjacent renal tissue, dissected to small segments, and placed in ice-cold Krebs solution. Arteries were prepared, and changes of isometric tension were recorded in myograph.

### Functional studies by organ bath

In the first series of experiments, two consecutive concentration–response curves to Phe were obtained in rings with endothelium. After the first concentration–response curve, rings were incubated with 100 μM NG-nitro-l-arginine methyl ester (L-NAME) or 3 μM 1H- [1, 2, 4] oxadiazolo[4,3-a]quinoxalin-1-one (ODQ) for 30 min. Contractions were determined as percentages of Emax obtained for the first concentration–response curve. At the end of each experiment, rings were collected, dried and weighed. Agonist-induced active tone was expressed as mN/mg tissue dry weight. In the second set of experiments, acetylcholine (ACh) was used to induce endothelial-dependent relaxation in phenylephrine (Phe, 1 μM)-contracted rings. The relaxant effects of sodium nitroprusside (SNP) were also examined. Each dilator was added cumulatively to bathing solution in 1-min intervals.

### Functional study by wire myograph

Renal interlobar arteries were initially stretched to an optimal resting tension (renal arteries: 3 mN) and equilibrated at 37 °C for 60 min before the start of experiments [14]. Rings were contracted with 60 mmol/L KCl and rinsed in Krebs solution. Each ring was then contracted by phenylephrine (Phe, 0.3 μM). Once a sustained tension was reached, ACh (3 nM-10 µM) was added cumulatively to evoke endothelium-dependent relaxations (EDRs).

### Flow-mediated dilatation (FMD) in pressure myograph

The third-order rat mesenteric artery (external diameter: 280–350 μm) was dissected free of surrounding adipose tissue and was cannulated between two glass cannulas in a chamber filled with 10 mL of oxygenated Krebs solution kept as previously described [15, 16]. The intraluminal pressure and vessel diameter were monitored by a light-inverted microscope (Zeiss Axiovert 40 microscope, model 11 P) with video camera, and the Myo-View software (Danish Myo Technology). Under no-flow condition, the artery segment was subjected to stepwise increment of 20 mmHg in intraluminal pressure from 20 to 80 mmHg at 5-min intervals at 37 °C and 3 μM phenylephrine (Phe) was added to induce vasoconstriction after the vessel’s diameter stabilized. FMD was triggered by pressure change that equals ~15 dynes/cm^2^ shear stress. Passive dilation was tested at the end of experiment in the Ca^2+^-free Krebs solution with 2 mM EGTA. FMD was calculated as the percentage of passive dilation: (flow-induced dilation—Phe tone)/(passive dilation—Phe tone).

### Western blotting analysis

Aortae were homogenized in tissue homogenizer on ice and centrifuged to collect supernatants. Protein samples (50 µg) were separated with 10 % SDS–PAGE and transferred to a nitrocellulose immobilon-P polyvinylidene difluoride membrane (Bio-Rad, Hercules, CA). Primary antibodies against eNOS (1:500, BD Transduction Laboratories), phosphorylated eNOS at Ser1177 (1:500, Abcam), NOX-2 (1:2000, Abcam), NOX-4 (1:500, Abcam), and GAPDH (1:10,000, Ambion) were used. The membranes were developed with an enhanced chemiluminescence detection system and exposed on X-ray films.

### Reactive oxygen species (ROS) determination

ROS measurement in *en face* endothelium and cross-sectional area of rat aortae was performed as described [16, 17]. Some aortae were freshly prepared for *en face* staining of ROS. Some aortae were embedded in OCT compound (Tissue-Tek), frozen in liquid nitrogen, and then cut into sections of 10-µm thickness on cryostat (Shandon). Fresh aortae or frozen sections of aortae were incubated for 20 min in 5 µM dihydroethidium (DHE; Molecular Probes)-containing PBS at 37 °C. Fluorescence was observed by Fluoview FV1000 laser scanning confocal system (Olympus, Tokyo, Japan; 515-nm excitation; 585-nm long-pass filter). DHE fluorescence intensity was analyzed by Fluoview FV10-ASW1.5 software. For each section, a square region with an area of 80 μm × 80 μm was selected for analysis. The summarized data represent the fold change in fluorescence intensity relative to that in control rat aortae [14].

### Determination of cGMP content

The concentrations of cGMP were measured using a commercial ELISA kit (Enzo Life Sciences, Inc. USA) as described before [18]. Aortae from sham control, OVX, BT-treated and E2-treated rats were incubated in a O_2_-saturated Krebs’ solution at 37 °C for 60 min and then exposed to 0.3 μM phenylephrine for 10 min and then to 1 μM ACh for 1 min. The aortae were removed, and the cGMP concentrations were determined.

### Drugs and chemicals

Phe, ACh, L-NAME, ODQ and SNP were purchased from Sigma-Aldrich. Stocks of drugs were kept at −20 °C.

### Data analysis

The relaxations were expressed as percentage reduction in Phe-induced contraction. The vasorelaxing effect was expressed as percentage reduction in evoked contraction. Concentration–relaxation curves were analyzed by nonlinear regression curve fitting using GraphPad Prism software (version 5.0) to estimate Emax as the maximal response and pD2 as the negative logarithm of the drug concentration that produced 50 % of Emax. In order to demonstrate the contribution of endothelial NO to Phe-induced tone, the first concentration–response curve (control) was subtracted from the second concentration–response curve (after inhibition of NO/cyclic GMP-dependent dilation) at individual concentrations for each ring. The amount of L-NAME or ODQ tone was compared among experimental groups as an index of the influence of basal NO. Data represent mean ± S.E.M. of rings from *n* animals. Concentration–response curves were analyzed by two-way ANOVA followed by Bonferroni post hoc tests. *P* < 0.05 indicates significant difference. For protein expression, the protein of interest was normalized to GAPDH and then expressed relative to control. Statistical significances of flow-mediated dilatation, protein of interest, and ROS measurement (Figs. 4, 5, and 6 respectively) were determined by one-way analysis of variance followed by Bonferroni’s multiple comparison test. Values of *P* < 0.05 were considered statistically significant.

## Results

### Chronic black tea consumption improved cholesterol profiles in OVX rats

17β-Estradiol serum levels were reduced in OVX rats compared with sham-operated control rats. However, chronic BT treatment did not significantly affect 17β-estradiol serum levels in OVX rats. Uterine weight decreased markedly in OVX rats, which was reversed by E2 treatment, while black tea consumption had no effects (Table 1). Body weight of OVX rats was increased, which was partially reversed by E2 treatment, but unaffected by chronic BT treatment. The ratio of heart weight over body weight was in OVX was decreased, but significantly increased in BT-treated and E2-treated groups. The OVX rats had higher serum levels of total cholesterol, triacylglycerols, HDL, non-HDL, and the non-HDL/HDL ratio than age-matched control rats. BT treatment modified the lipid profile in OVX rats through lowering total cholesterol, triglyceride, and HDL-C, while E2 treatment only affected total cholesterol and HDL-C. Both BT and E2 treatments did not change the non-HDL/HDL ratio in OVX rats.

**Table 1:** Serum 17β-estradiol levels, aortic cyclic GMP (cGMP) concentration, serum lipid profiles, body weight (BW), heart weight (HW), and uterine weight (UW) of control, OVX rats, and OVX rats receiving administration of black tea (OVX + BT) or estrogen treatment (OVX + E_2_)

Parameters	Control	OVX	OVX + BT	OVX + E_2_
17β-Estradiol (pg/mL)	52.3 ± 7.3	7.9 ± 2.3^a,^***	10.1 ± 3.7^a,^***	38.2 ± 2.8^b,^***^; c,^***
cGMP (pmol/mg protein)	2.65 ± 0.33	0.89 ± 0.15^a,^***	2.11 ± 0.41^b,^*	2.68 ± 0.69^b,^*
Total cholesterol (mg/dL)	78.87 ± 2.16	112.50 ± 4.04^a,^***	98.45 ± 1.50^ a,^***^; b,^**	96.48 ± 4.59^a,^**^; b,^*
Triglyceride (mg/dL)	55.89 ± 3.00	79.33 ± 8.93^a,^*	42.95 ± 1.53^a,^**^; b,^**	66.31 ± 7.13^c,^**
HDL-C (mg/dL)	56.02 ± 1.82	74.72 ± 2.98^a,^***	66.27 ± 0.59^a,^***^; b,^*	46.2 ± 2.56^a,^*^; b,^***^; c,^***
Non-HDL-C (mg/dL)	21.02 ± 1.50	38.43 ± 5.69^a,^*	32.87 ± 2.39^a,^**	39.49 ± 4.08^a,^**
Non-HDL-C:HDL	0.27 ± 0.02	0.58 ± 0.09^a,^**	0.50 ± 0.05^a,^**	0.48 ± 0.05^a,^**
BW (g)	255.6 ± 1.8	346.1 ± 7.5^a,^***	336.1 ± 3.7^a,^***	280.6 ± 5.5^a,^***^; b,^***^; c,^***
HW (g)	1.07 ± 0.02	1.09 ± 0.02	1.14 ± 0.03	1.06 ± 0.02^c,^***
% HW/BW	0.43 ± 0.01	0.31 ± 0.02^a,^***	0.38 ± 0.01^a,^**^; b,^**	0.39 ± 0.01^a,^*^; b,^**
UW (g)	0.67 ± 0.02	0.11 ± 0.04^a,^***	0.10 ± 0.01^a,^***	0.330 ± 0.03^a,^***^; b,^***^; c,^***
UW/BW	0.26 ± 0.01	0.02 ± 0.00^a,^***	0.03 ± 0.00^a,^***	0.12 ± 0.01^a,^***^; b,^***^; c,^***

Results are mean ± SEM of 6–9 rats. Statistical significance versus control (a), versus OVX (b) or versus OVX + BT (c) is indicated by * *P* < 0.05, ** *P* < 0.005 and *** *P* < 0.001

**Fig. 1:**
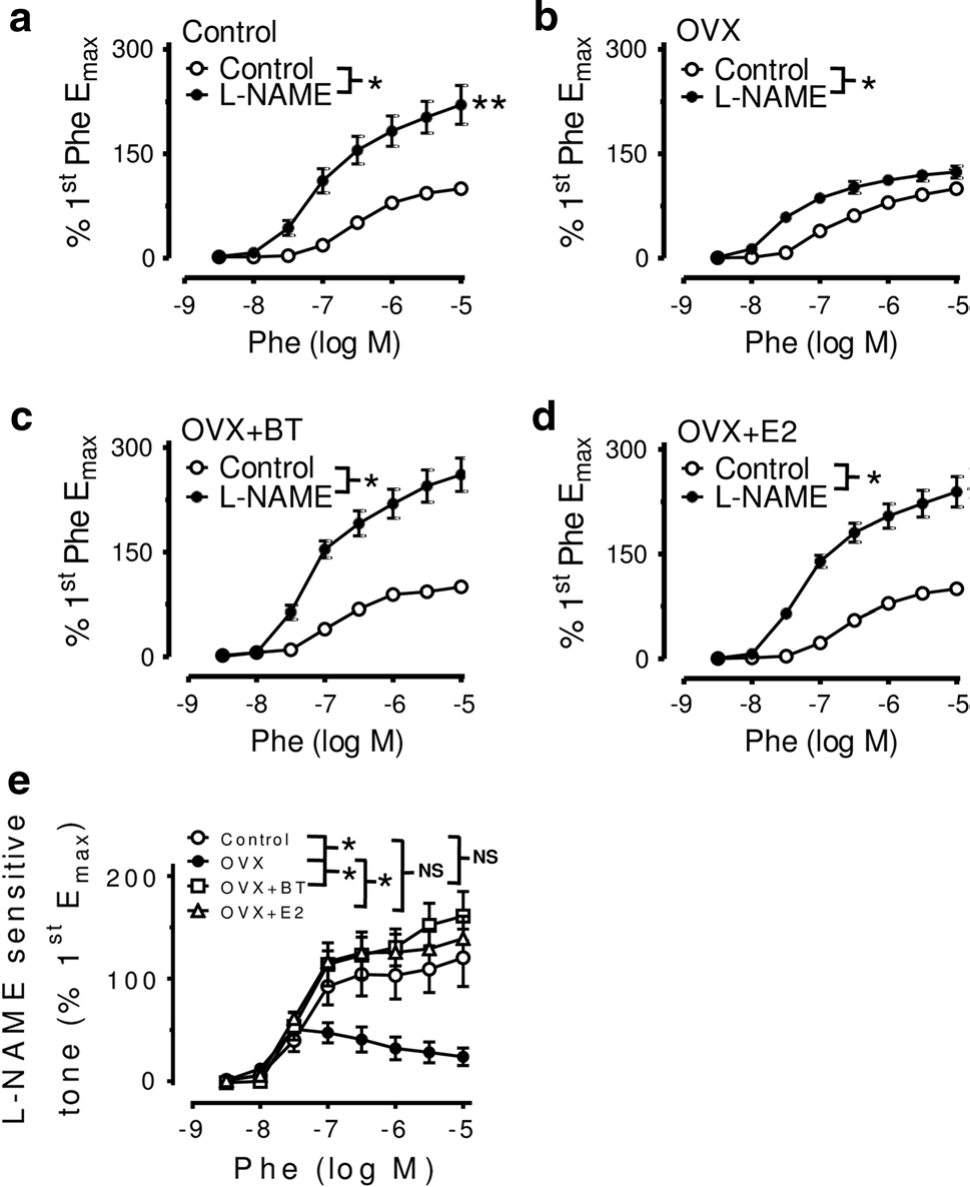
**a** Concentration–response curves for phenylephrine obtained in rings from **a** control, **b** ovariectomized (OVX) and **c** black tea (BT)-treated OVX (OVX + BT) and **d** estrogen (E2)-treated (OVX + E) rats in the absence and presence of L-NAME. Rings were exposed to L-NAME for 30 min before repeating the second concentration–response curve. **e** The L-NAME-sensitive component of phenylephrine-induced contractions was determined by subtracting the first (control, without L-NAME) from the second (with 100 μM L-NAME) concentration–response curve at each data point in aortic rings with endothelium from Control, OVX, OVX + BT rats and OVX + E2. Statistical difference between curves is indicated (*P < 0.05, two-way ANOVA). Data are mean ± S.E.M. of seven rats in each group. NS stands for non-significant

BT or estrogen treatment reduced phenylephrine-induced contraction through an increase in NO bioavailability treatment with L-NAME (100 μM) enhanced Phe-induced contractions in aortic rings with endothelium from all groups. Aortic rings from control (Fig. 1a), BT-treated (Fig. 1c) and E2-treated OVX rats (Fig. 1d) exhibited a greater augmentation of the Phe-induced contraction after inhibition of NO synthase compared with those from OVX rats (Fig. 1b). The Phe-induced contraction was greater in aortae from OVX rats when compared with control and BT/E2-treated rats (1st CRC: Emax: 0.66 ± 0.07 g/mg dry weight in control, 1.29 ± 0.1 g/mg dry weight in OVX, 0.54 ± 0.06 g/mg dry weight in OVX + BT, 0.69 ± 0.04 g/mg dry weight in OVX + E2, *n* = 7, *P* < 0.0001). After L-NAME treatment, there are significant differences in the Emax for Phe on aortas from OVX rats when compared with control and BT-treated rats only, not in E2-treated rats (2nd CRC: Emax: 1.26 ± 0.12 g/mg dry weight in control, 1.61 ± 0.09 g/mg dry weight in OVX, 1.28 ± 0.07 g/mg dry weight in OVX + BT, 1.47 ± 0.1 g/mg dry weight in OVX + E2, *n* = 7, *P* < 0.05). No differences in pD2 values were found among control, OVX, OVX + BT and OVX + E2. The L-NAME-sensitive tone was higher in rings in control and BT- or E2-treated OVX rats than that in OVX rats (Fig. 1e).

**Fig. 2:**
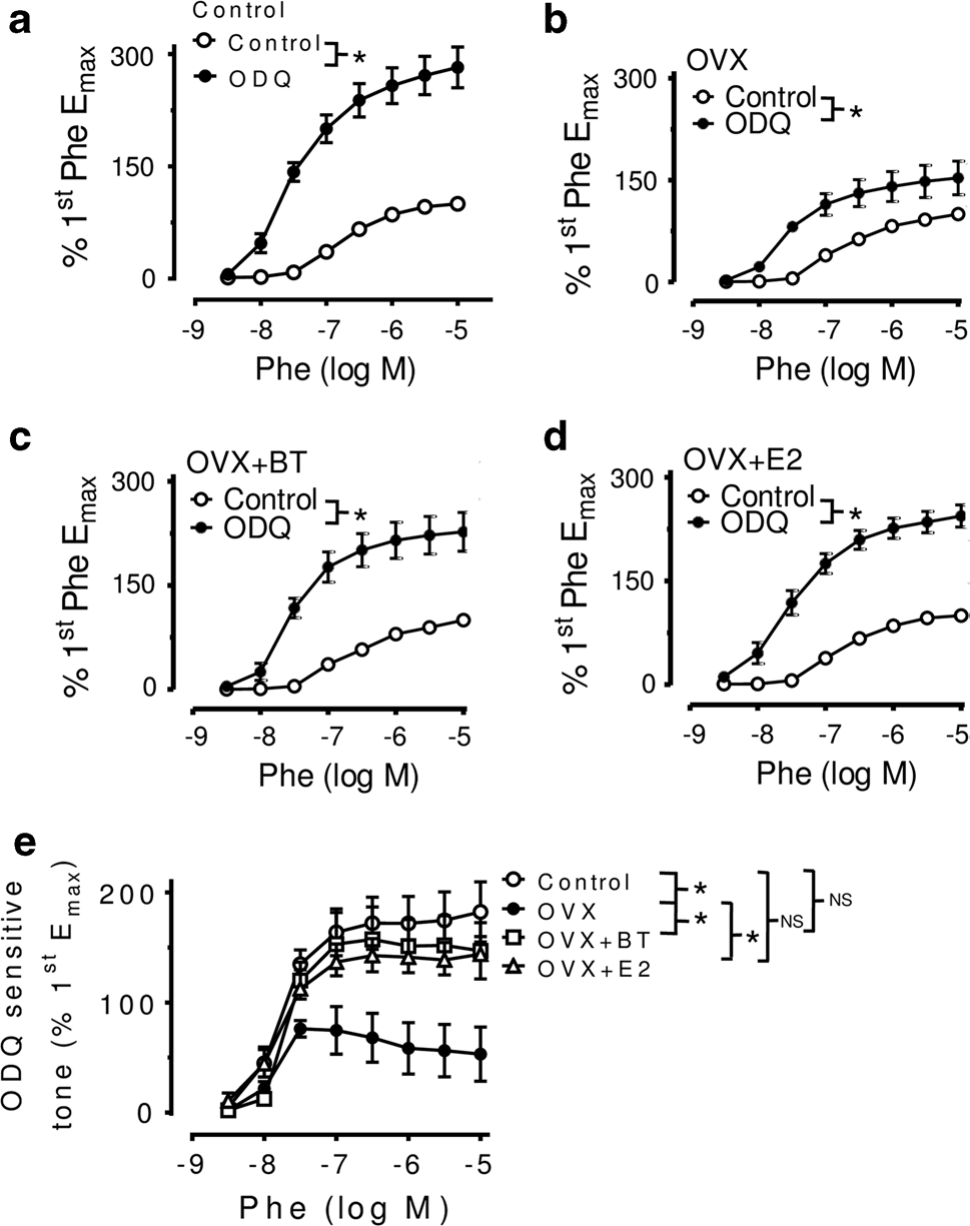
Concentration–response curves (CRC) for phenylephrine obtained in aortic rings from **a** Control, **b** OVX, **c** OVX + BT, **d** OVX + E2 in the absence and presence of ODQ. Rings were exposed to 3 μM ODQ for 30 min before repeating the second CRC. **e** ODQ-sensitive component of phenylephrine-induced contractions was determined by subtracting the first (control, without ODQ) from the second (with 3 μM ODQ) CRC at each data point in rings with endothelium from Control, OVX, OVX + BT rats and OVX + E2. Statistical difference between curves is indicated (*P < 0.05, two-way ANOVA). Data are mean ± S.E.M. of 5–7 rats in each group. NS stands for non-significant

Likewise, treatment with ODQ (3 μM, an inhibitor of NO-dependent soluble guanylate cyclase) was less effective in enhancing the Phe-induced responses in rings from OVX rats (Fig. 2b) compared with those from control (Fig. 2a) or BT- or E2-treated OVX rats (Fig. 2c, d). The ODQ-sensitive tone was higher in aortae in control and BT- or E2-treated OVX rats than those in non-treated OVX rats (Fig. 2e). Further, cGMP levels after ACh stimulation were reduced in the aortae from OVX rats compared with those from control rats. ACh-stimulated cGMP production was significantly increased in the aortas from BT- and E2-treated rats compared with those from OVX rats (Table 1).

### Black tea treatment improved endothelial functions in OVX rats

**Fig. 3:**
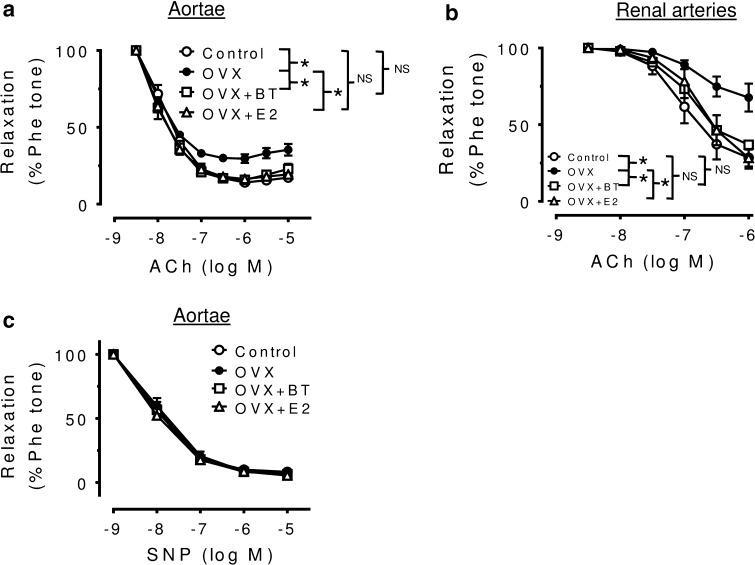
Black tea improves EDRs in aortae and renal arteries of OVX rats. **a** Summarized graphs show that black tea and estrogen improved EDRs in aortae and renal arteries (**b**) of OVX rats. Comparable endothelium-independent relaxations to SNP were shown in aortae (**c**) from four groups. Statistical difference between curves is indicated (*P < 0.05, two-way ANOVA). Data are mean ± S.E.M. of nine rats in each group. NS stands for non-significant

Ovariectomy significantly impaired the acetylcholine-induced maximal relaxation in aortae (Emax: 68.2. ± 8.53 %, *P* < 0.05 compared to 84.7 ± 9.48 % obtained in control rats). EDRs in response to accumulative concentration of ACh in Phe-precontracted segments of aortae were improved by BT and E2 treatments in OVX rats (Emax: 68.2 ± 8.53 % in OVX, 81.8 ± 7.45 % in OVX + BT, 82.5 ± 13.61 % in OVX + E2, *n* = 9, *P* < 0.0001) (Fig. 3a) without affecting the SNP-induced relaxations (Fig. 3c). On the other hand, no differences in pD2 values were found among control, OVX, OVX + BT and OVX + E2 aortas (7.91 ± 0.12; 8.06 ± 0.13; 8.14 ± 0.21; 8.15 ± 0.19, respectively).

**Fig. 4:**
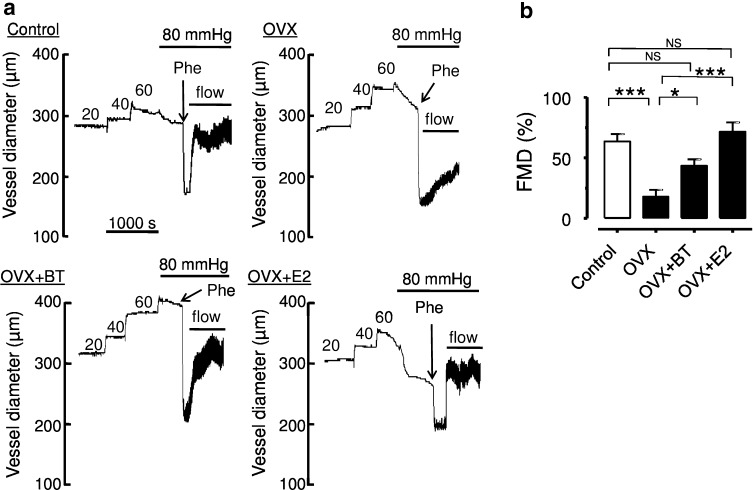
Effects of black tea and estrogen treatments on flow-mediated dilation (FMD) in isolated small mesenteric artery segments. Representative traces (**a**) and data summary (**b**) showing the effect of black tea and estrogen on flow-mediated dilation. Data are mean ± S.E.M. of 10–12 rats in each group. Data were compared by one-way ANOVA followed by Bonferroni’s multiple comparison test (*P < 0.05, ***P < 0.0001, and NS stands for non-significant)

Likewise, ovariectomy significantly impaired the ACh-induced maximal relaxation in renal arteries (Emax: 34.4 ± 1.5 %, *P* < 0.05 compared to 73.7 ± 1.8 % obtained in control rats). BT and E2 treatments improved EDRs in renal arteries (Emax: 34.4 ± 1.5 % in OVX, 66.7 ± 2.0 % in OVX + BT, 77.9 ± 1.7 % in OVX + E2, *n* = 9, *P* < 0.0001; Fig. 3b). There was no difference in pD2 values were found among control, OVX, OVX + BT and OVX + E in renal arteries (7.02 ± 0.14; 6.78 ± 0.23; 6.89 ± 0.11; 6.73 ± 0.16, respectively). The flow-induced relaxations in third order of mesenteric arteries were also improved significantly by BT and E2 treatments in OVX rats (Fig. 4a, b & c).

### Black tea consumption increased phosphorylated eNOS and reduced NAD(P)H oxidase-mediated oxidative stress

**Fig. 5:**
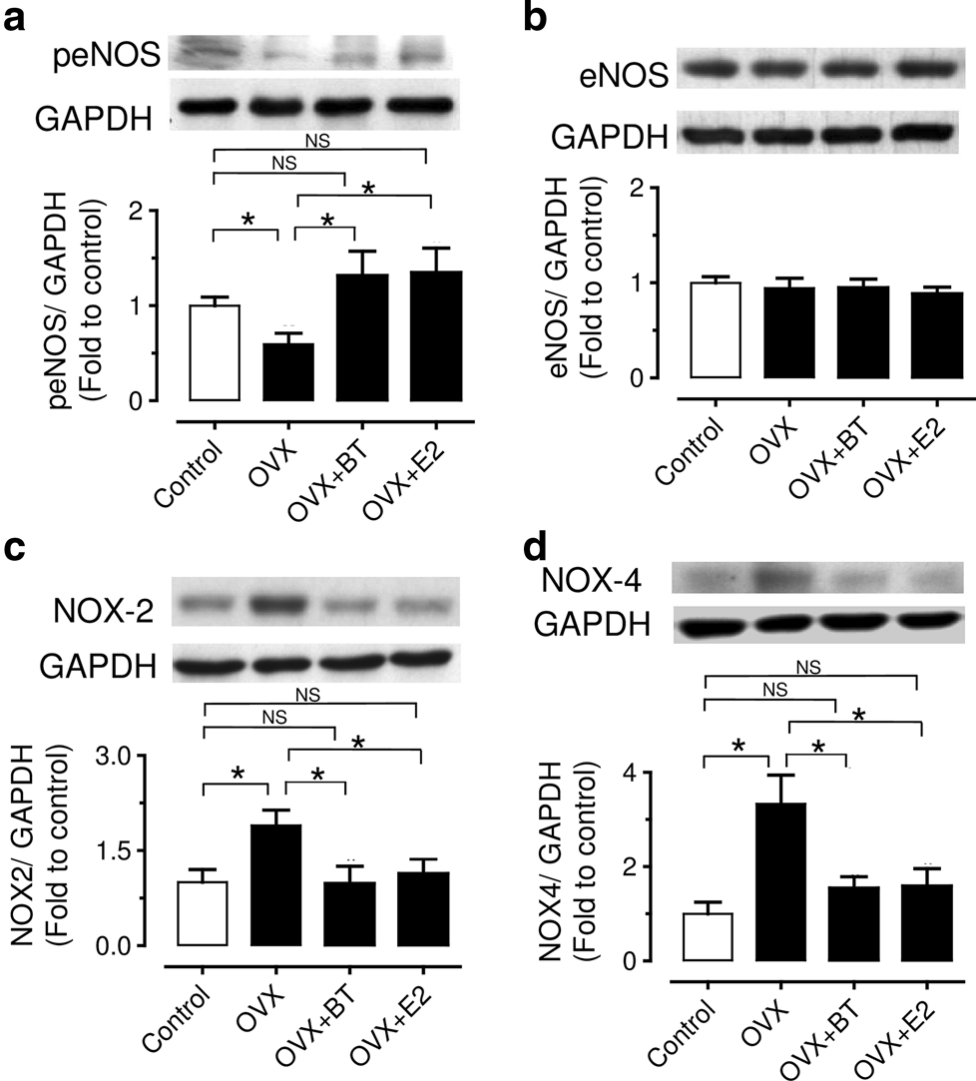
Effects of chronic black tea and estrogen treatments on the levels of total eNOS (**a**), phosphorylated eNOS, **b** NOX-2, **c** NOX-4. Results are mean ± S.E.M. of 6–8 rats. Intensities were normalized to GAP(D)H and expressed relative to control. Data were compared by one-way ANOVA followed by Bonferroni’s multiple comparison test (*P < 0.05 and NS stands for non-significant)

Chronic BT treatment to OVX rats augmented the phosphorylation of eNOS (Fig. 5a) without altering the total eNOS (Fig. 5b). Western blotting results showed that BT treatment reversed the elevated protein levels of membrane-bound NAD(P)H oxidase subunits, NOX2 (Fig. 5c) and NOX4 in aortae of OVX rats (Fig. 5d).

### Black tea attenuated ROS production in vivo

**Fig. 6:**
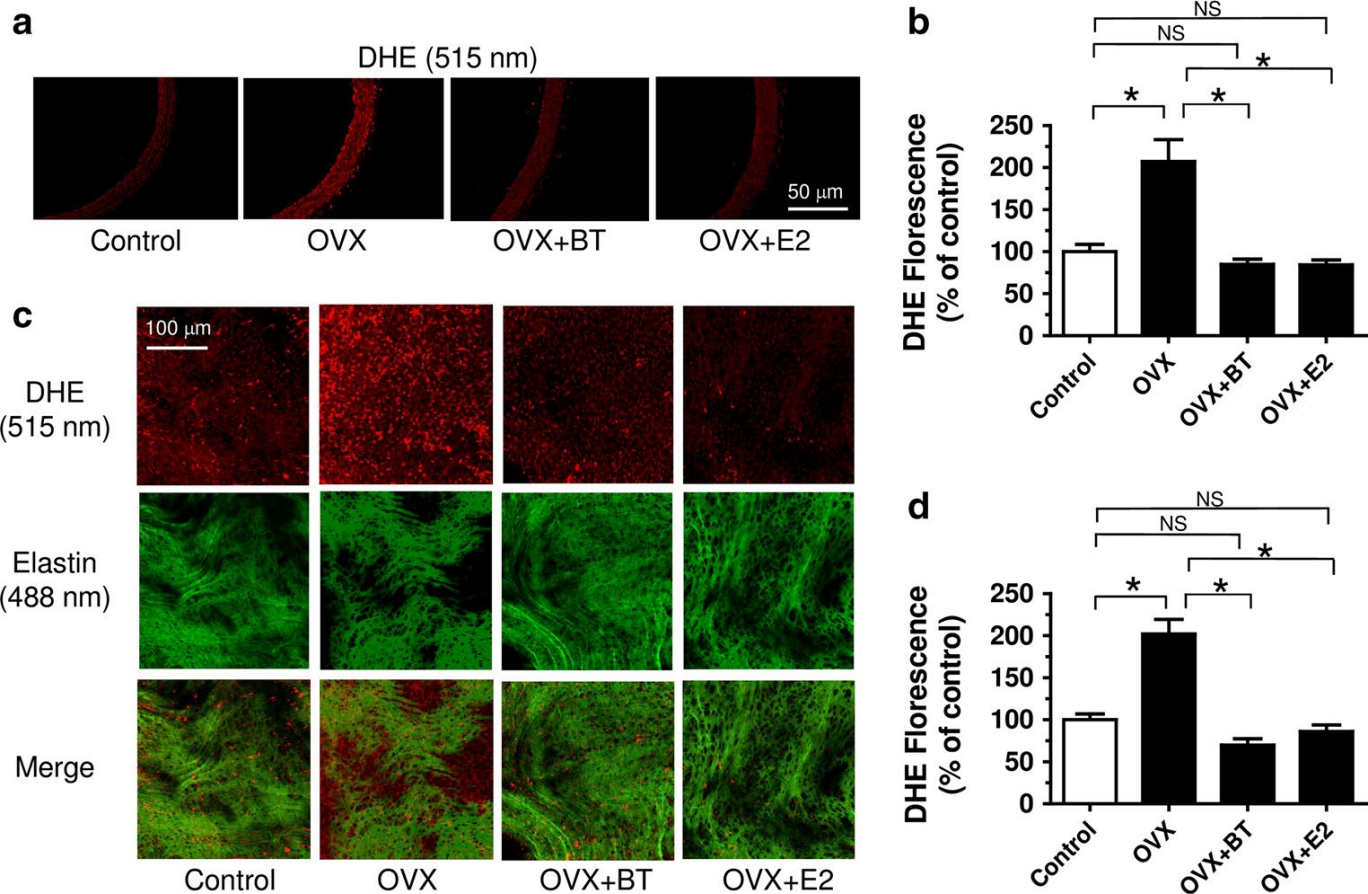
Black tea treatment reduced reactive oxygen species (ROS) over-production in the endothelium of aorta from OVX rats. Representative images and summarized data showing **a**, **b** of cross sections and **c**, **d** en face endothelium of control, OVX, OVX + BT and OVX + E2 rats. *Red*, DHE fluorescence (excitation: 515 nm) in the nucleus; *green*, autofluorescence of elastin underneath the endothelium (excitation, 488 nm). Results are mean ± S.E.M. of nine rats in each group. Data were compared by one-way ANOVA followed by Bonferroni’s multiple comparison test (*P < 0.05 and NS stands for non-significant)

Elevation in ROS levels was reduced following BT and E2 treatment in aortae (both *en face* endothelium and cross-sectional area) from OVX rats (Fig. 6).

## Discussion

The present study demonstrates that a 4-week treatment with BT restores the impaired EDRs in renal arteries and aortae of OVT rats. The flow-mediated dilatations in the third-order mesenteric arteries were also augmented in OVX rats receiving BT treatment. These effects are likely associated with an increase in NO bioavailability. We showed for the first time that black tea treatment reduces oxidative stress in aortas of OVX rats, which is associated with the inhibition of NAD(P)H oxidase. The elevated production of ROS, especially superoxide anions is known to inactivate NO, thus to impair endothelial function. Taken together, the present study reveals that black tea has the potential to induce endothelial adaptations similar to estrogen, primarily those involving NO availability probably inhibiting oxidative stress in estrogen deficiency without affecting estrogen level.

The clinical importance of the endothelial NO pathway is well documented. Endothelial dysfunction, showed as reduced NO levels, is one of the most common pathological changes in cardiovascular diseases and is revealed by impaired endothelium-dependent vasodilatation or by enhanced vasoconstriction [19]. Both human and animal studies suggest diminished NO bioactivity after menopause [20]. NO is derived from its precursor, l-arginine, and its synthesis can be blocked by l-arginine analogues, such as L-NAME. Once released from the endothelial cells, NO diffuses toward the underlying vascular smooth muscle cells and stimulates the cyclic GMP-dependent pathway leading to relaxation that is sensitive to ODQ (an inhibitor of soluble guanylate cyclase). Therefore, the differences in basal NO release can be determined indirectly by differences in the contractile responses to a vasoconstrictor before and after treatment with L-NAME or ODQ. The present study demonstrated that aortae from control or BT-treated and E2-treated OVX rats exhibited a greater potentiation of phenylephrine-evoked contractions in the presence of L-NAME (Fig. 1) or ODQ (Fig. 2) as compared with OVX rats. The present data indicate that the basal release of NO is augmented significantly in OVX rats receiving BT and E2 treatment.

Indeed, ingestion of BT led to significant increments in brachial artery flow-mediated, i.e., NO-mediated, dilation (FMD). Duffy et al. [21] also demonstrated that both acute and prolonged black tea ingestion significantly improved FMD in patients with coronary artery disease [21]. The beneficial effects of tea on FMD were clearly related to significant increments in plasma flavonoid concentration, thereby strongly supporting the conclusion that black tea consumption markedly improved NO bioavailability in vivo because of its high flavonoid content. In a randomized controlled parallel study, Hodgson et al. [22] showed a significant and consistent increase in endothelium-dependent vasodilation after regular consumption of 5 cups/day (250 mL each) of black tea, given over a period of 4 week in mildly dyslipidemic subjects. The present study suggests that BT and E2 treatment is highly effective to reverse endothelial dysfunction in conduit arteries such as aortae and renal arteries (Fig. 3) and the flow-mediated dilatation in small resistance mesenteric arteries (Fig. 4) of OVX rats. The results of this study suggest that black tea has comparable effects to estrogen treatment in restoring NO availability and, in a similar way, the endothelial dysfunction observed in the animal model of estrogen deficiency.

An improved contribution of the NO pathway following BT treatment may involve either stimulation of eNOS activity or greater expression of the eNOS protein. The present finding showed that BT treatment failed to affect the expression of eNOS protein, however, induced the phosphorylation of eNOS (Fig. 5a, b). In addition, the increase in NO bioavailability might be attributed to the reduction in ROS production. Accumulative evidences suggested that ROS production during estrogen deprivation may lead to the development of endothelial dysfunction [23–25], especially those ROS derived from NADPH oxidase, which is the main source of superoxide anion in the vascular system [26]. The present results suggest that BT and E2 treatment suppressed the expression of NADPH oxidase such as NOX-2 and NOX-4 (Fig. 5c, d) and thereby reduced the oxidative stress in vascular wall of OVX rats (Fig. 6). Taken together, the present novel finding reveals that black tea improves endothelial function in the same way as estrogen through inhibiting oxidative stress and increasing NO bioavailability in OVX rats.

The cholesterol levels are generally high in postmenopausal women [27]. In the present experiment, ovariectomy resulted in an increase in total and HDL cholesterol, which was reversed by estrogen treatment in line with the findings in previous studies [14, 28]. Estrogen therapy is shown to induce favorable changes in plasma lipid profiles [29]. Although HDL is generally recognized as beneficial to endothelial function, the effect of estrogen may be partially due to reduced total cholesterol level which is not related to modulation of HDL level. A clinical trial by Davies et al. [30] demonstrated that daily inclusion of black tea reduces total cholesterol level significantly in mildly hypercholesterolemic adults and, therefore, minimizes the risk of coronary heart disease. We also present evidence supporting the effectiveness of black tea and estrogen treatments in modulating the lipid profiles in an estrogen deficient state OVX rats (Table 1). These findings may have important implications for the treatment of postmenopausal cardiovascular complications.

In conclusion, the present study indicates that chronic oral administration with black tea, similar to E2 treatment. Improved NO bioavailability accompanied by the reduced ROS production through the down-regulation of the expression of NADPH oxidase. The present data suggest that postmenopausal women can benefit from regular black tea consumption, probably counteracting the development of cardiovascular and metabolic abnormalities related to the rise in the level of the cholesterol level.
